# Reprogramming of the Caseinolytic Protease by ADEP Antibiotics: Molecular Mechanism, Cellular Consequences, Therapeutic Potential

**DOI:** 10.3389/fmolb.2021.690902

**Published:** 2021-05-13

**Authors:** Heike Brötz-Oesterhelt, Andreas Vorbach

**Affiliations:** ^1^ Microbial Bioactive Compounds, Interfaculty Institute of Microbiology and Infection Medicine, University of Tuebingen, Tübingen, Germany; ^2^ Cluster of Excellence: Controlling Microbes to Fight Infection, Tübingen, Germany

**Keywords:** ClpP, protease, acyldepsipeptide, antibiotic, mode of action, conformational control, drug discovery

## Abstract

Rising antibiotic resistance urgently calls for the discovery and evaluation of novel antibiotic classes and unique antibiotic targets. The caseinolytic protease Clp emerged as an unprecedented target for antibiotic therapy 15 years ago when it was observed that natural product-derived acyldepsipeptide antibiotics (ADEP) dysregulated its proteolytic core ClpP towards destructive proteolysis in bacterial cells. A substantial database has accumulated since on the interaction of ADEP with ClpP, which is comprehensively compiled in this review. On the molecular level, we describe the conformational control that ADEP exerts over ClpP, the nature of the protein substrates degraded, and the emerging structure-activity-relationship of the ADEP compound class. On the physiological level, we review the multi-faceted antibacterial mechanism, species-dependent killing modes, the activity against carcinogenic cells, and the therapeutic potential of the compound class.

## Physiological Functions and Operation Mode of the Clp Protease in Various Organisms

The caseinolytic protease Clp is ubiquitous in prokaryotes and prokaryote-derived organelles of eukaryotic cells (Yu and Houry, 2007). Clp is important for protein turnover and homeostasis in a broad range of species and particularly relevant under protein stress conditions, such as elevated temperatures, exposure to protein damaging agents, or during the stationary phase (Frees et al., 2014). Regulatory proteolysis is the second major task of Clp, i.e., the rapid and coordinated degradation of central regulatory proteins, often transcription factors, to control differentiation and development programs of bacterial and eukaryotic cells (Nagpal et al., 2013; Mahmoud and Chien, 2018; Nouri et al., 2020). In the well-studied model organism *Bacillus subtilis*, the Clp protease regulates, e.g., the heat-shock response, sporulation, natural genetic competence, and swarming motility (Frees et al., 2014; Elsholz et al., 2017). In many pathogenic bacterial species, Clp is essential for the expression of critical virulence factors. Clp deletion mutants of, e.g., *Staphylococcus aureus*, *Streptococcus pneumoniae*, *Listeria monocytogenes,* and *Enterococcus faecalis* were impaired in host survival, intracellular persistence, or biofilm formation (Brötz-Oesterhelt and Sass, 2014; Bhandari et al., 2018; Moreno-Cinos et al., 2019a). Also, in prokaryote-derived organelles, the Clp protease has essential functions. In mitochondria, Clp is required for, e.g., reducing protein stress, proper functioning of respiratory chain complexes, the regulation of mitophagy, and fission/fusion dynamics (Bhandari et al., 2018; Nouri et al., 2020). In chloroplasts, the Clp protease is essential for plastid biogenesis and plant survival and in *Plasmodium falciparum* for apicoplast biogenesis and survival of the malaria parasite (Kim et al., 2009; Nishimura and van Wijk, 2015; Florentin et al., 2020).

A functional, compartmentalized Clp protease complex consists of the tetradecameric proteolytic core ClpP and a cognate hexameric Clp-ATPase (Baker and Sauer, 2012). Two stacked heptameric ClpP rings form the secluded proteolytic chamber, which can only be accessed through small apical pores. Fourteen active sites, generally containing the canonical catalytic triad (Ser, His, Asp) of a typical serine protease, reside at the equatorial plane of the chamber (Zeiler et al., 2013). ClpP alone can only hydrolyze peptides; the interaction with a Clp-ATPase is mandatory for the degradation of proteins (Maurizi et al., 1990). The catalytic sites themselves have very limited substrate preference, and substrate specificity is achieved by restricted substrate access to the chamber. Clp/HSP100 enzymes, which belong to the group of ATPases associated with diverse cellular activities (AAA+ ATPases), recognize substrates destined for degradation by ClpP via certain degradation tags (degrons), phosphorylation of dedicated arginine residues, or general physicochemical properties (i.e., denatured regions). Then, the Clp-ATPases unfold the substrate protein in a process fueled by ATP hydrolysis and accompanied by translocation of the polypeptide chain through a ClpP pore (Baker and Sauer, 2012; Olivares et al., 2014; Trentini et al., 2016). Adaptor proteins can mediate the interaction of a substrate to a Clp-ATPase and stabilize the active conformation of the latter. The presence of dedicated adaptors for specific substrates allows fine-tuning of regulatory proteolysis. Besides, elaborate feedback loops exist, in which either adaptors are themselves recognized as Clp protease substrates or Clp-ATPases, in a ClpP-independent chaperone function, protect substrates from Clp protease degradation (Kirstein et al., 2009b). Recently, it was also detected that the ribosome-associated trigger factor chaperone Tig modulates substrate degradation rates of ClpXP (Rizzolo et al., 2021).

Depending on the species, bacterial genomes generally encode either a single ClpP homolog (e.g., *B. subtilis*, *S. aureus*, or *E. coli*) (Yu and Houry, 2007), or two ClpP homologs (e.g., *Mycobacterium spec.*, *Listeria monocytogenes* or *Chlamydia trachomatis*) (Akopian et al., 2012; Zeiler et al., 2013; Pan et al., 2019) and only rarely more than two (e.g., *Streptomyces* spec.) (Viala et al., 2000). In cases where two *clpP* isoforms exist, the formation of mixed ClpP1P2 hetero-tetradecamers is common. In such a heteromeric complex, each homolog assembles into a separate heptamer, both of which stack to build the hetero-tetradecameric barrel. Often, such assembly leads to catalytic stimulation (Akopian et al., 2012; Zeiler et al., 2013; Pan et al., 2019). In most bacterial species, the ClpP proteolytic core interacts with two or three different Clp-ATPases, which have distinct substrate preferences (Baker and Sauer, 2012; Frees et al., 2014). Considering organelles, mitochondria contain a relatively reduced system that consists of a single ClpP isoform and ClpX as the sole ATPase, whereas chloroplasts possess an exceptionally complex Clp machinery, with multiple ClpP homologs plus catalytically inactive regulatory ClpP isoforms even mixing within the heptameric rings (Nishimura and van Wijk, 2015; Bhandari et al., 2018).

**FIGURE 1:**
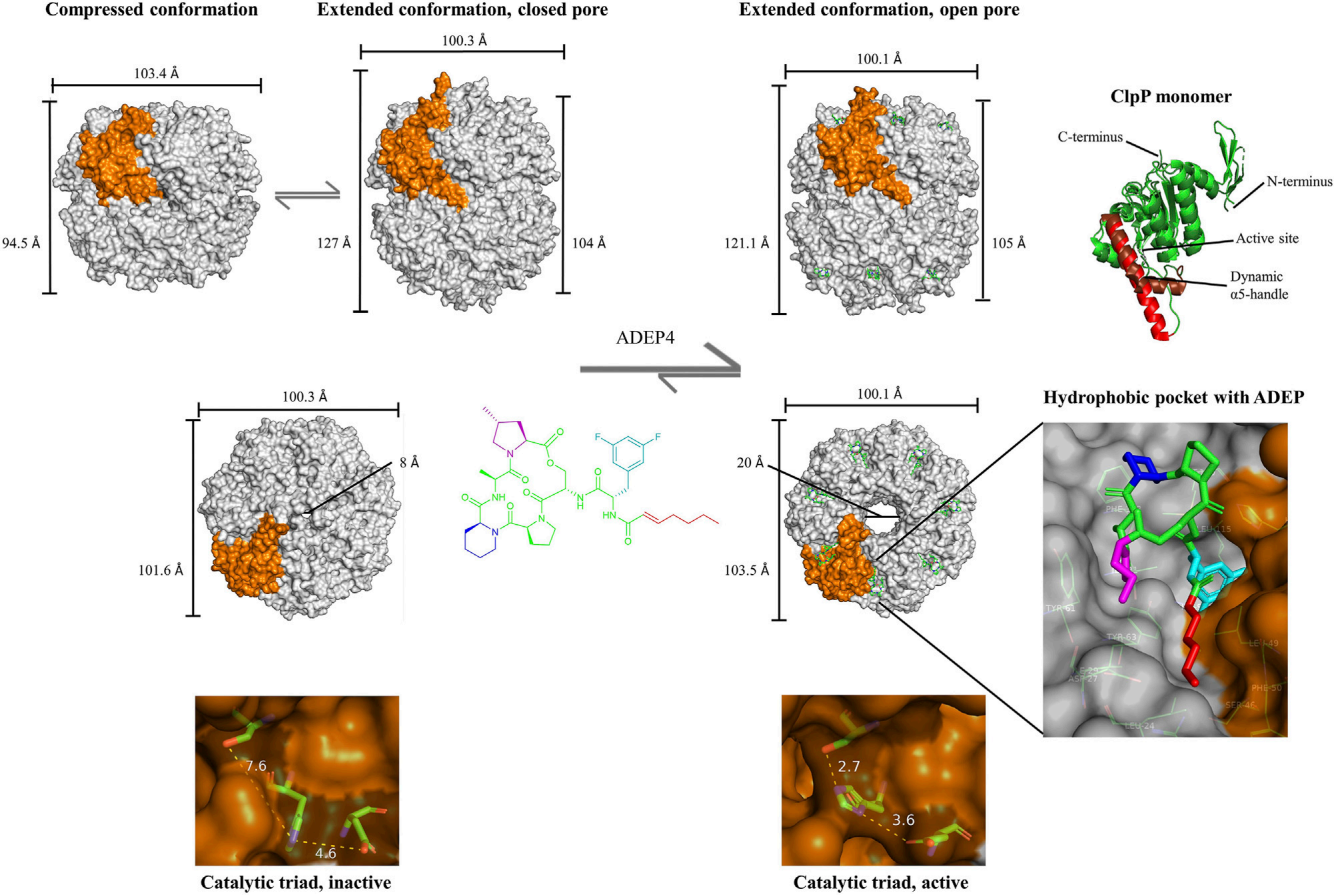
Impact of ADEP on ClpP at the molecular level. Conformational dynamics of ClpP and conformational control exerted by ADEP. Upper row, side view on the tetradecameric barrel of ClpP, switching between the “compressed” and “extended” state as the two endpoints of the conformational transition. The term “compact” is used for an intermediate state (not shown). ADEP stabilizes the extended, active conformation. A single ClpP protomer within the barrel is highlighted in orange. Single protomer showing the dynamic α5 helix (right). Middle, top view on the apical surface of ClpP, depicting the closed pore in *apo*ClpP and the widened pore upon ADEP4 binding. Magnified H-pocket formed by two neighboring protomers and occupancy by ADEP4. Bottom, magnified catalytic triads of the compressed, inactive *vs.* extended, active conformation. In the nucleophilic attack of the catalytic serine on the peptide bond carbonyl, the serine hydroxyl proton is abstracted by the histidine imidazole, and the positive charge at the histidine is stabilized by the carboxy group of the aspartate. The hydrogen network between the catalytic triad is essential for the interaction and the optimal distance for a hydrogen bond ranges from 2.7 to 3.3 Å. *S. aureus* ClpP structures are shown: Compressed barrel (PDB code: 4EMM) (Ye et al., 2013); compressed monomer (PDB code: 3QWD) (Geiger et al., 2011), extended barrel with closed (PDB code: 6TTY) and open pore (PDB code: 6TTZ) (Malik et al., 2020).

The Clp protease is a dynamic molecular machine. In crystal structures, ClpP appeared in different barrel conformations designated as “compressed”, “compact”, and “extended” states (Geiger et al., 2011; Zhang et al., 2011; Liu et al., 2014). Compressed and extended are considered the two endpoints of a dynamic transition and the compact state a stable intermediate (Malik and Brötz-Oesterhelt, 2017). In the compressed conformation, ClpP is about 10–15 Å shorter than in the extended state and also wider, and there is only loose contact between the heptameric rings because in the so-called “handle-region” the α5 helix that is crucial for establishing the inter-ring interactions is kinked in all monomers (Figure 1). In the extended conformation, the α5 helices are stretched, and they establish a hydrogen bond network between the two rings (Gersch et al., 2013). Important for catalysis, the active site is located at the intersection of α5 and the main ClpP body, and the conformation of the catalytic triad is changed upon α5 movement (Geiger et al., 2011; Zhang et al., 2011; Gersch et al., 2013). Only in the extended conformation, the three active site residues are in the correct distance and spatial orientation to form hydrogen bonds, an interaction required for peptide hydrolysis. The flexibility of the handle region seems also to be important for the formation of transient side pores at the ring interface to facilitate product release after hydrolysis (Sprangers et al., 2005; Lee et al., 2011). Formation of a disulfide bridge in the α5 helix of a ClpP mutant strongly restricted conformational movement of the handle region and led to peptide accumulation in the proteolytic chamber (Sprangers et al., 2005). Another flexible domain of the ClpP protomer is the N-terminal loop. The seven N-terminal loops of a heptamer ring flank an entrance pore, and the dynamics of the N-terminal domain regulate pore diameter and thereby substrate access (Alexopoulos et al., 2012; Vahidi et al., 2018; Mabanglo et al., 2019; Malik et al., 2020). Each apical face of ClpP presents seven cavities lined with hydrophobic amino acids, which serve as docking points for the Clp-ATPases. Each cavity, termed “hydrophobic pocket” (H-pocket), is formed from two protomers within the same heptameric ring, and amino acids from adjacent monomers jointly line it (Baker and Sauer, 2012). Long flexible loops extending from the body of the Clp-ATPase recognize the H-pockets via a conserved three-amino acid motif (V/IGF/L) positioned at the tip of the “IGF-loops”, and this interaction stabilizes the active, extended conformation of ClpP (Kim et al., 2001). Clp-ATPases are hexamers, while the interacting surface of ClpP is heptameric. The molecular reason for this mismatch has long been elusive. Recent cryo-EM structures of Clp-ATPase/ClpP co-structures from several organisms revealed highly flexible interactions between the two partners mismatched in symmetry. It seems that the IGF-loops engage with different H-pockets over time. The structural plasticity at the Clp-ATPase/ClpP interface seems essential to enable the dynamics relevant to substrate unfolding, translocation, hydrolysis, and product release (Gatsogiannis et al., 2019; Ripstein et al., 2020a; Fei et al., 2020). The exact nature of the Clp-ATPase motion at the ClpP interface is subject of current research (Tsai and Hill 2020). While one model proposed a slow rotation of ClpX relative to ClpP (Ripstein et al., 2020a), another model favored ClpX dynamics without rotation (Fei et al., 2020; Kim et al., 2020).

The Clp protease system is a complex and elaborate biological machinery, from the perspective of its molecular interactions and dynamics as well as the versatility of functions and relevance to the lifestyle of numerous organisms. Also, there is broad evidence that the Clp protease is an excellent anti-virulence, antibiotic and anticancer target. Over the last 2 decades, a vast amount of information has been published on these topics, of which the introductory passage of the current review can only provide a glimpse. For deeper insight, the interested reader is referred to the reviews cited above. In the following passages of this review, we will focus on the class of acyldepsipeptide (ADEP) compounds that first demonstrated that Clp could serve as a druggable target.

## Dysregulation of the Bacterial Clp Protease by Acyldepsipeptide Antibiotics

### Acyldepsipeptide in Its Natural Producer Strain *Streptomyces hawaiiensis*

**FIGURE 2:**
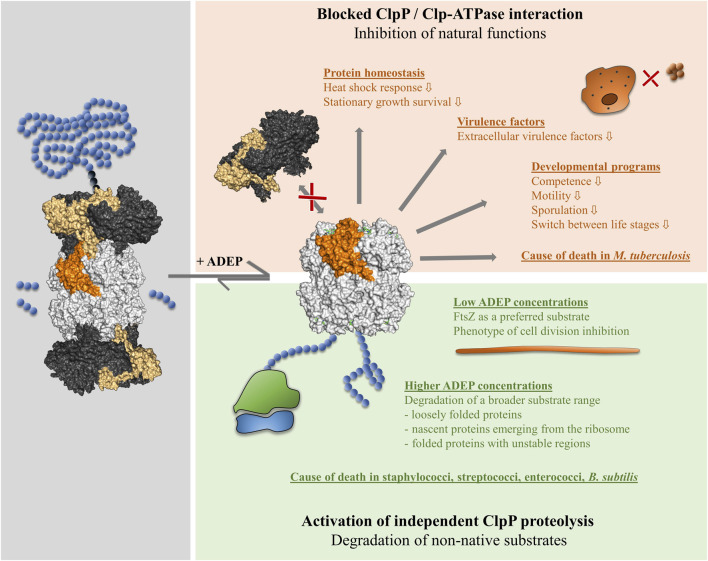
Impact of ADEP on ClpP on the physiological level. ADEP acts by a dual mechanism. The binding of ADEP to the hydrophobic pockets at the apical surface of ClpP causes rapid and efficient displacement of all cooperating Clp-ATPases (red box). Consequently, all the natural functions of the Clp protease in protein homeostasis and regulatory proteolysis are inhibited, of which examples are given. In *M. tuberculosis*, which depends on a functioning Clp protease for survival, the blocked Clp-ATPase/ClpP interaction is the cause of death. Conformational control of ClpP by ADEP bestows independent proteolytic capabilities to the ClpP core (green box). A variety of non-native substrates are untimely degraded in a concentration-dependent manner, of which examples are given. The indicated members of the Firmicutes and other bacterial species die by self-digestion.

The ADEP natural product complex A54556 is produced by *Streptomyces hawaiiensis* NRRL15010 and comprises several closely related congeners. All derivatives contain a macrolactone core composed of five amino acids (Ser, Pro, *N*-MeAla, Ala, Pro/MePro) and a polyene side-chain of varying length connected to the core by a phenylalanine linker. The initially proposed structure (Michel and Kastner 1985) was later slightly revised (Hinzen et al., 2006). ADEP 1 (factor A, Figure 2) emerged as the most active among the main components of the natural product complex, displaying a Minimal Inhibitory Concentration (MIC) of 6 μg/ml against *S. aureus* (Brötz-Oesterhelt et al., 2005), and it became the ADEP prototype and progenitor of an extended class of synthetic derivatives with improved properties. ADEP1 was reliably produced under all media conditions tested and seems to be produced during the entire life cycle of *S. hawaiiensis* (Thomy et al., 2019). Characterization of the biosynthetic gene cluster identified two non-ribosomal peptide synthetases (NRPS) for the assembly of the amino acid chain and cyclization, genes responsible for the biosynthesis of the characteristic methyl-proline moiety and a type II polyketide synthase (PKS) for the production of the polyene side-chain. An additional *clpP* gene is located close to the biosynthetic genes. Its product, termed ClpP_ADEP_, functions as an ADEP resistance factor and was sufficient to provide high-level resistance to all *Streptomyces* species tested when heterologously expressed (Thomy et al., 2019). Six congeners initially detected in the A54556 natural product complex were also prepared by total synthesis and tested for their antibacterial activities *in vitro* (Goodreid et al., 2014). ADEP1 was confirmed as the most active among the main components of the natural product complex. Synthesized Compound 4 (Goodreid et al., 2014), corresponding to one of the minor components originally named “factor D” (Michel and Kastner, 1985), demonstrated an 8-fold lower MIC against *S. aureus* and also lower MIC values against *S. pneumoniae* and *Neisseria meningitidis* (Goodreid et al., 2014). Pharmacological evaluation of ADEP1 demonstrated promising antibacterial activity against Gram-positive bacteria, including multi-resistant isolates of *S. aureus*, *S. pneumoniae*, and enterococci (Brötz-Oesterhelt et al., 2005), but also liabilities, such as limited stability to light (due to the polyene side-chain), low metabolic stability and limited solubility. Several research teams have worked on and succeeded in the total synthesis of synthetic ADEP congeners with substantially improved properties (see *Structure Activity Relationship of the Acyldepsipeptide Class*).

### Proteolytic Activation of ClpP by Conformational Control

The target of ADEP is ClpP, a fact confirmed in a range of different bacterial species. Initial studies were performed with the natural product ADEP1, and more potent synthetic congeners were shown to have the same mode of action. ADEP is a hydrophobic molecule that binds to the same H-pocket that is typically addressed by the Clp-ATPases (Lee et al., 2010; Li et al., 2010). The affinities of improved ADEPs for ClpP are in the low µM or even sub-µM range (e.g., Goodreid et al., 2016; Griffith et al., 2019; Malik et al., 2020).

ADEP establishes contacts to both neighboring ClpP protomers forming the H-pocket (Figure 1), and these interactions support the intra-heptamer stability (Lee et al., 2010; Li et al., 2010). However, the stabilizing potential of ADEP goes beyond promoting the contacts within the same ring. For instance, *B. subtilis* ClpP, which is often purified in the monomeric state, assembles rapidly into a tetradecameric barrel upon ADEP addition (Kirstein et al., 2009a; Lee et al., 2010), and purified human mitochondrial ClpP shifts from the heptameric to the tetradecameric state (Lowth et al., 2012). Thermal shift assays demonstrated that ADEP binding enhances the overall folding stability of *S. aureus* ClpP (Gersch et al., 2015). Hydrogen–deuterium exchange experiments using *E. coli* ClpP showed that ADEP strongly enhances the rigidity of the handle region (Sowole et al., 2013), and an *S. aureus* ClpP mutant (D172N) trapped in the inactive compressed state, shifted to the active extended state when ADEP was bound (Gersch et al., 2015). A modeling study offered a rational explanation as to why ADEP stabilizes ClpP in the extended state (Zhang et al., 2011). While ADEP fits very well into the H-pocket conformation presented in crystal structures of extended ClpP, there is a strong steric clash of the ADEP side-chain with the H-pocket in the compressed conformation. Consequently, the extended conformation is favored upon ADEP binding, and the compound shifts the dynamic equilibrium of ClpP to the extended conformation (Figure 1). As noted above, the active sites are in the catalytically competent arrangement only when the α5 helix is extended. This fact explains how ADEP can activate catalysis allosterically. In experiments where ADEP and β-lactone suicide inhibitors were applied in combination, ADEP was shown to increase the acylation and deacylation rates of ClpP (Gersch et al., 2015).

There is elaborate cross-talk in ClpP through long-distance conformational relays, first, along the vertical axis between the H-pocket and the active site and second, horizontally between the H-pocket and the N-terminal domain lining the entrance pore (Malik and Brötz-Oesterhelt, 2017). With the exception of human mitochondrial ClpP, which presented itself in a compact state despite ADEP binding (Wong et al., 2018), all ADEP-ClpP co-crystal structures reported to date show ClpP in the extended conformation with a widened entrance pore. This is the case for *B. subtilis* ClpP (Lee et al., 2010), *E. coli* ClpP (Li et al., 2010; Mabanglo et al., 2019), *S. aureus* ClpP (Vahidi et al., 2018; Griffith et al., 2019; Malik et al., 2020), *Mycobacterium tuberculosis* ClpP (Schmitz et al., 2014b; Vahidi et al., 2020), *Neisseria meningitidis* ClpP (Mabanglo et al., 2019), and *Enterococcus faecium* ClpP (Brown-Gandt et al., 2018). A critical residue for regulating the conformation of the N-terminal gate is a conserved tyrosine (e.g., Y63 in *S. aureus* and *B. subtilis* ClpP) within the H-pocket. ADEP establishes hydrogen bonds to this residue and rotates it by 90°, resulting in a domino effect that triggers pore expansion by 10–15 Å (Lee et al., 2010; Li et al., 2010; Ni et al., 2016; Stahl and Sieber, 2017). Mutating the tyrosine to alanine has the same effect (Ni et al., 2016). In the process of pore opening, the electrostatic interaction network at the ClpP entrance pores is reorganized, the normally flexible N-terminal loops lining the pore uniformly adopt an ordered β-hairpin conformation, and the entire N-terminal domain slightly moves outward (Li et al., 2010; Alexopoulos et al., 2012; Schmitz et al., 2014b; Mabanglo et al., 2019; Malik et al., 2020). The fact that ADEP binding to the H-pocket controls the conformation of the entire ClpP barrel is impressively visible in the ADEP-bound structure of *M. tuberculosis* ClpP (Schmitz et al., 2014b). Here, the tetradecamer consists of one ClpP1 ring and one ClpP2 ring, and ADEP targets exclusively ClpP2. Accordingly, the crystal structure shows ADEP occupancy only in the ClpP2 H-pockets, while the ClpP1 pockets are empty. Nonetheless, both pores are wide open, emphasizing that ADEP engagement with the ClpP2 H-pocket controls the entire barrel and opens the opposite pore 90 Å away (Schmitz et al., 2014b). In the different ClpP structures so far determined, the diameter of ClpP pores widened by ADEP is in the range of 20–30 Å, sufficient for the passage of one to two α-helices of a protein substrate. While ClpP can usually only degrade small peptides, it is capable of degrading proteins in the ADEP-activated state (Brötz-Oesterhelt et al., 2005). The nature of the substrates is discussed in *Substrates of Acyldepsipeptide-Activated ClpP*.

The fact that ADEP shifts the equilibrium of ClpP to the extended conformation was also demonstrated for an unusually distorted and catalytically inactive *S. aureus* ClpP mutant. The V7A mutation in the N-terminal region of ClpP triggered an asymmetrical split-ring conformation, with protomers no longer being in the same plane and one protomer presenting a kinked α5 helix, which resulted in prominent equatorial side-pores (Vahidi et al., 2018; Ripstein et al., 2020b). Even such a strong distortion was cured by ADEP addition, and catalytic function was restored. A cryo-EM density map of the ADEP-bound V7A mutant showed all protomers in the same plane and the extended conformation, lacking side-pores (Vahidi et al., 2018). In the light of such a strong ordering effect, the question emerges how dynamic ADEP-bound ClpP is and how degradation products can leave the proteolytic chamber. It might well be that ADEP-activated ClpP is less dynamic than a ClpP barrel operated by a Clp-ATPase, which can place its distinct IGF-loops into the H-pockets in a sequential and rhythmic manner. There is also no indication that ADEP can generate an active force to push the substrate into the proteolytic chamber. All the ADEP-ClpP crystal structures reported to date and also the cryo-EM structure of V7A showed full occupancy of all H-pockets by ADEP. During crystallization, saturating activator concentrations are used, while during a proteolytic *in vitro* assay in solution or within an ADEP-exposed cell, the activator might diffuse on and off. Lower activator concentrations leave single H-pockets temporarily unoccupied. If this might influence the overall conformation of ClpP, potentially leading to transient side-pores, has not been investigated. However, it was noted that sub-stoichiometric ADEP concentrations negatively impacted the peptide hydrolysis rate of *S. aureus* ClpP (Gersch et al., 2015). Regrading product release, it should also be considered that the apical pores of ADEP-bound ClpP are wide open, generating a suitable peptide exit route, and that ADEP-activated ClpP operates with lower processivity than a Clp-ATPase/ClpP complex, as explained below.

### Steric Hindrance of the ClpP–Clp-ATPase Interaction

Concerning the structural considerations discussed in the previous section, it should be emphasized that while ADEP activates the proteolytic core ClpP for independent activity, it simultaneously inhibits all the natural functions that ClpP performs typically in conjunction with Clp-ATPases (Kirstein et al., 2009a). ADEP binds to the same position that is normally recognized by the IGF-loops of the Clp-ATPases (Lee et al., 2010; Li et al., 2010) and efficiently prevents the interaction of ClpP with its cognate Clp-ATPase partners (Figure 2). The binding of only a single ADEP molecule to one hydrophobic pocket at one apical surface of the homo-tetradecameric *S. aureus* ClpP was sufficient to reduce ClpXP hydrolysis by half (Gersch et al., 2015). ADEP also disassembled a preformed complex consisting of *B. subtilis* ClpCP-MecA, with MecA being an adaptor of ClpC (Kirstein et al., 2009a). In a kinetic study with *E. coli* ClpXP, an extremely rapid dissociation of the Clp-ATPase from ClpP was observed upon ADEP addition (ClpXP half-life approx. 1s in the presence of ADEP). This happened despite the presence of ATP, which is known to stabilize the ClpP-Clp-ATPase interactions (Amor et al., 2016). Also, here, a single ADEP molecule was sufficient to make an entire *E. coli* ClpX ring dissociate from ClpP, and ATP hydrolysis was not required for this to occur. A “dynamic competition” model was proposed. Interactions of Clp-ATPase IGF-loops with the ClpP H-pockets are transient, with individual IGF loops temporarily unbinding while others remain bound. ADEP rapidly fills the vacant H-pocket, and a steric clash of the compound with the transiently unbound IGF-loop would be a plausible explanation for the dramatic destabilization of the complex (Amor et al., 2016). Interference of ADEP with Clp-ATPase-mediated protein substrate degradation by ClpP *in vitro* was demonstrated across species for all Clp-ATPases tested to date (e.g., Kirstein et al., 2009a; Lee et al., 2010; Carney et al., 2014b; Schmitz et al., 2014b; Gersch et al., 2015; Amor et al., 2016; Famulla et al., 2016).

### Substrates of Acyldepsipeptide-Activated ClpP

ADEP binding to ClpP results in a dual mode of action. Blocking the interaction of ClpP with its cooperating Clp-ATPases leads to the accumulation of native substrates and a lack of Clp protease function in protein homeostasis and regulatory proteolysis (Figure 2). Simultaneously, the constitutive activation of ClpP results in an uncontrolled degradation of non-native substrates (Brötz-Oesterhelt et al., 2005; Kirstein et al., 2009a). *In vitro*, the model substrate casein is rapidly degraded by ADEP-activated ClpP, whereas it is stable for more than 24 h in the sole presence of ClpP (Brötz-Oesterhelt et al., 2005). While ClpP is highly processive, when working together with its cognate Clp-ATPases, ADEP-activated ClpP is not. In concert with a Clp-ATPase, the protein substrate remains engaged with the Clp-ATPase/ClpP protease complex and is inserted into the ClpP pore in an uninterrupted process generated by the dynamic motion of the Clp-ATPase on the apical surface of ClpP (Ripstein et al., 2020a). Only short peptides leave the degradation chamber. In contrast, a range of larger and smaller casein fragments emerges during degradation by ADEP-activated ClpP, demonstrating reduced processivity (Brötz-Oesterhelt et al., 2005; Kirstein et al., 2009a). Whether the smaller fragments result from the repeated engagement of a pre-digested larger fragment or whether the different fragment sizes reflect a different duration of a single engagement event is unknown.

Casein is a loosely folded protein, which makes its entry into the ADEP-widened pore and subsequent hydrolysis plausible (Figure 2). To compare the impact of ADEP-activated ClpP on further model substrate proteins with a higher and lower degree and speed of folding, an *in vitro* transcription/translation system was employed, using actively processing *vs.* stalled ribosomes (Kirstein et al., 2009a). The results were consistent and confirmed by pulse-labeling and immunoblotting experiments in intact *E. coli* cells. ADEP-activated ClpP attacked nascent polypeptide chains as they emerged from the exit tunnel of ribosomes in the process of translation (Figure 2). Not all substrates were equally susceptible. The slower the folding kinetics, the higher the susceptibility to degradation. Proteins capable of adopting a stable fold after release from the ribosome were susceptible during ongoing translation but resisted degradation once translation was completed and the protein released and folded. After removal of the total protein fraction from bacterial cells by trichloroacetic acid precipitation, ADEP-treated bacterial cells showed a substantial increase in the global percentage of (poly)peptides (i.e., fragments) too small for precipitation (Kirstein et al., 2009a). Whole-cell proteomic analysis of actively growing *B. subtilis* revealed a strong heat shock response indicative of protein stress as well as the presence of many N-terminal truncation products of pulse-labeled proteins synthesized during ADEP exposure (Brötz-Oesterhelt et al., 2005).

Proteome analysis was also conducted in stationary *S. aureus* cells treated for 24 h at 10-fold the minimal inhibitory concentration (10xMIC) of ADEP, and a proteolytic attack on several hundreds of protein species was noted (Conlon et al., 2013). The ADEP-activated ClpP core has broad destructive potential. Although the experimental set-up did not allow differentiation between the extent of co-translational *vs.* potential post-translational degradation (i.e., whether the fragments were generated from nascent chains or folded proteins), the fact that the ADEP-activated ClpP core had such a broad destructive potential on resting cells suggests that also folded proteins can be attacked (Conlon et al., 2013). This has important implications for the therapeutic prospect of the ADEP class of compounds as most other known antibiotics affect only growing cells (Conlon et al., 2013).

The ADEP-widened N-terminal gates of ClpP are well suited for the entry of nascent polypeptide chains or unfolded protein regions. To which extent the degradation of *folded* proteins is possible is an area of ongoing research. *In vitro* degradation assays using ADEP-activated ClpP were conducted with several purified proteins (DnaK, Tig, GroEL), selected as putative substrates as they had been detected in N-terminally truncated forms in ADEP treated cells. Besides, several known substrates (MecA, McsB, ComK, Spx) of natural Clp protease complexes (i.e., ClpP in cooperation with a Clp-ATPase) were chosen. All of these proteins resisted the degradation *in vitro* (Kistein et al., 2009). To date, there is only a single mature folded protein, which has been reported to be rapidly and efficiently degraded by ClpP at low ADEP concentrations *in vitro*, and this is the cell division pacemaker protein FtsZ (Figure 2). The fact that FtsZ is particularly sensitive to proteolysis by ADEP-activated ClpP was observed already some time ago when it was noted that bacterial cells treated with low ADEP concentrations close to the MIC retained substantial biosynthetic capacity and developed a prominent phenotype of cell division inhibition (Sass et al., 2011). Rapidly after ADEP addition, FtsZ disappeared from the cytoplasm of ADEP-treated *B. subtilis*, as shown by immunoblotting, and ClpP was necessary and sufficient for FtsZ degradation, while Clp-ATPases were not required (Sass et al., 2011).

Recently, the molecular basis for the exceptional sensitivity of the folded FtsZ protein was discovered (Silber et al., 2020). Contrary to initial expectations, it is not the extended, unfolded C-terminus that makes FtsZ such a good substrate for ADEP-activated ClpP but rather the physicochemical and structural characteristics of the N-terminal domain. The degradation process involves the short N-terminus of FtsZ, which extends only slightly beyond the body of the globular protein. The flexible portion of the N-terminus is definitively too short for reaching the active sites of ClpP when projected through an ADEP-widened pore. Nonetheless, the N-terminus makes an important contribution, as deletion of the first ten amino acids of *B. subtilis* ClpP abolished the characteristic degradation sensitivity of FtsZ (Silber et al., 2020). According to the current model, the short N-terminus of *B. subtilis* FtsZ is important for establishing a stable interaction with ADEP-activated ClpP and its hydrophobicity is instrumental in binding to the hydrophobic ClpP pore. Although ADEP cannot actively “push” a substrate into the opened pore of ClpP, in contrast to a Clp-ATPase, physicochemical interactions between the substrate and the widened ClpP pore probably influence the duration and strength of substrate engagement. The second decisive characteristic of the FtsZ-GTPase is a previously unnoted conformational flexibility of its folded N-terminal domain when neither GTP nor GDP is bound. Engaging ClpP further destabilizes the N-terminal domain of the nucleotide-free FtsZ. Unfolding is promoted, α-helices previously embedded in the folded N-terminal domain are exposed and become vulnerable to proteolytic attack, followed by degradation of the entire protein (Silber et al., 2020). This process occurs at an equimolar ratio of ADEP to ClpP monomer. It was also noted that at a 2.5 molar excess of ADEP over ClpP, the C-terminus of FtsZ becomes a second target site. The molecular explanation for this differential targeting process and whether it might involve different levels of conformational control of ADEP over ClpP is still elusive (Silber et al., 2020).

### Bacterial Cell Biology During Acyldepsipeptide Exposure

ClpP is most probably the only target of ADEP in Firmicutes because *B. subtilis*, *S. aureus*, *Streptococcus pneumoniae* and *Enterococcus* sp. carrying deletions or loss-of-function mutations in ClpP showed high-level resistance to ADEP (Malik et al., 2020). The same was shown for *E. coli* (Brötz-Oesterhelt et al., 2005) and probably applies to other bacterial species, too. Despite acting on this single target, ADEP treatment results in different phenotypes depending on the compound concentration applied. At low ADEP concentrations (1–2x MIC), *B. subtilis* cells retain a remarkable capacity to produce biomass and develop into extremely long filaments. *S. aureus* and *S. pneumoniae* swell to several times the volume of the untreated control (Sass et al., 2011; Mayer et al., 2019). Although it is likely that ADEP-activated ClpP also attacks further proteins in addition to FtsZ under these conditions, degradation of the major cell division protein results in a particularly prominent phenotype across species and the most obvious kind of damage. Cell growth can go on for hours under such careful treatment conditions. The cell morphology of *B. subtilis* was monitored at the single-cell level by time-lapse microscopy during both ADEP exposure and recovery after compound removal. After 2 h in the presence of ADEP at 1–2x MIC, most *B. subtilis* cells could still recover. They synthesized new FtsZ and resumed cell division during recovery in ADEP-free medium (Mayer et al., 2019). Even after 5 h of treatment and reaching a length of 100–200 μm, many filaments still displayed regular nucleoid segregation. Then, rather rapidly, a point of no return was reached for most cells, beyond which they failed to recover. It was noted that the extremely long ADEP-induced filaments were particularly prone to lysis, an effect that had been described before for filaments generated by FtsZ knockdown (Beall and Lutkenhaus, 1991). The situation could be aggravated by the fact that ClpP has natural functions in regulating the cell envelope metabolism (Frees et al., 2014), which can no longer be performed in the presence of ADEP.

Notably, the phenotype of extreme filamentation (rods) and extensive swelling (cocci) did only develop at ADEP concentrations close to the MIC. At tenfold higher concentrations (10–12x MIC), biomass production ceased rapidly; rods remained relatively short, cocci small, and the number of colony-forming units decreased (Mayer et al., 2019). Obviously, additional damage is afflicted to the cells as ADEP levels rise, most probably through the degradation of further protein species that now become substrates. The observation that different regions of FtsZ can be targeted by ClpP at a higher molar surplus of ADEP over ClpP (see *Substrates of Acyldepsipeptide-Activated ClpP*) suggests a concentration-dependent attack on different protein species when the entire proteome is considered. Time-kill experiments showed that killing of *S. aureus* can be achieved with similar efficiency either by prolonged exposure (several hours) to ADEP concentrations close to the MIC or short-term exposure (10 min) to very high ADEP concentrations. However, applying 16x MIC for only 1 h was clearly not enough, and the cells could resume growth in fresh medium after a lag period of about 2 h (Mayer et al., 2019). It is likely that differences in the substrate spectrum of ADEP-activated ClpP underlie the uncommon biphasic response.

The fact that ADEP application to Firmicutes leads to rapid FtsZ degradation is also instrumental in studying the cell division process. ADEP application and removal can be performed rapidly, which is particularly beneficial in time-lapse experiments. Furthermore, the ADEP-triggered “chemical FtsZ knockdown” can be easily combined with other genetically induced cell division mutations. The finding that ADEP-activated ClpP preferably targets FtsZ in the nucleotide-free state at low ADEP concentrations (see *Substrates of Acyldepsipeptide-Activated ClpP*) was exploited in a recent study on FtsZ ring formation and progression in *B. subtilis* (Silber et al., 2021). ADEP primarily leads to the depletion of nucleotide-free monomeric FtsZ from the cytoplasmic pool, which is required for FtsZ ring dynamics. The study showed that newly formed FtsZ rings rapidly disappeared after ADEP addition, demonstrating their dependence on the cytoplasmic, nucleotide-free monomeric FtsZ pool and the dynamics of the FtsZ ring. In contrast, mature FtsZ rings, marked by arrival of the peptidoglycan synthases to the division site, were stable and capable of concluding the cell division cycle. The result suggests that the dynamics of FtsZ ring play a minor role in the late stages of divisome progression (Silber et al., 2021).

### Activities of Acyldepsipeptide in Different Bacterial Species

The ADEP class, and especially several modified congeners, obtained by total synthesis and deviating from the original natural products, proved highly effective against Firmicutes, including multidrug-resistant isolates of pathogenic species (Brötz-Oesterhelt et al., 2005; Brown-Gandt et al., 2018; Mroue et al., 2019). MICs in the sub-µM and even nM range were observed against *S. aureus* (including methicillin-resistant strains, MRSA), *S. pneumoniae* (including penicillin-resistant strains, PRSP), *Enterococcus faecium*, and *Enterococcus faecalis* (including vancomycin-resistant strains, VRE) (Brötz-Oesterhelt et al., 2005; Carney et al., 2014b; Goodreid et al., 2016; Griffith et al., 2019). The killing mode in this group of organisms is the degradation of non-native substrates by the ADEP-activated ClpP core (Figure 2). This conclusion can be clearly drawn from the fact that *clpP* is not essential in these bacteria during growth in the laboratory under MIC assay conditions (Msadek et al., 1998; Malik et al., 2020). In all of the species mentioned, FtsZ seems to be a preferential target, as all of them displayed a phenotype of cell division inhibition at ADEP-concentrations close to the MIC (Sass et al., 2011).

**FIGURE 3:**
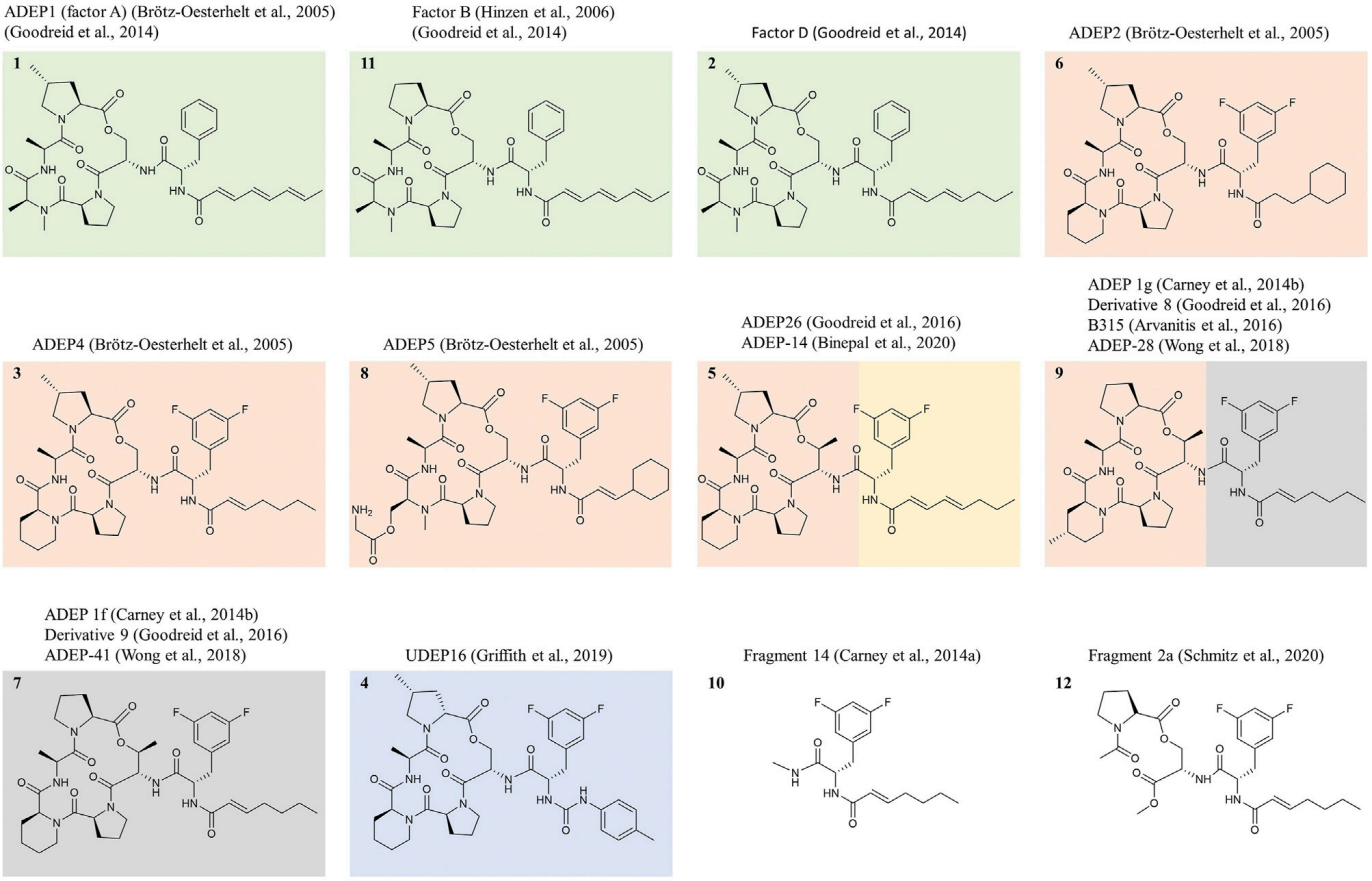
Prominent ADEP congeners discussed in this review and structure-activity-relationship. Exemplary ADEP congeners are depicted that were prominently featured in the scientific literature on the compound class over the last 15 years. Compounds only described in patents are not shown. Natural products are shown in green boxes, and all other depicted structures were obtained by total synthesis. The natural product ADEP1 (“factor A”) represents the progenitor of the compound class, while “factor B” lacks the MePro and is 4-fold less active against *S. aureus*. Compounds synthesized originally to improve the activities against staphylococci, streptococci, and enterococci are shown in red boxes. ADEP2, ADEP4, and ADEP5 originate from an initial optimization campaign directed at improving the activity against *S. aureus* and increasing chemical and metabolic stability. Bis-fluorination of Phe led to strongly enhanced antibacterial activity. Rigidification of the macrolactone core by exchanging *N*-MeAla for pipecolic acid increased activity further. Reduction of the number of double bonds in the side-chain increased stability. Removal of the two terminal double bonds was sufficient to prevent sensitivity to light and temperature. This modification also increased metabolic stability, although ADEP4 was still a high-clearance drug. Removal of the α,β-double bond in ADEP2 enhanced metabolic stability further but led to a loss in antibacterial activity. ADEP2 was a medium clearance drug and still highly active but less active than ADEP4. ADEP5 illustrates that *N*-MeAla allows the attachment of bulkier moieties. ADEP5 has a substantially higher solubility. Further rigidification of the macrocycle by replacing Ser for *allo-*Thr brought an additional increase in antibacterial activity. Introduction of a urea moiety into the side-chain allowed to omit the α,β-double bond without loss of antibacterial activity. Within the ureadepsipeptide series (blue box), metabolic stability is markedly improved. ADEP26 (ADEP-14) showed very good activity against *Neisseria* (anti-Neisseria activity indicated by a yellow box). Against *Neisseria* and *E. coli*, the multiple-unsaturated side-chain is superior to the mono-unsaturated one. The same was observed for *Streptomyces*, against which ADEP1 proved superior to ADEP4. ADEP-28 (ADEP 1g, ADEP B315) and ADEP-41 (ADEP 1f) were featured as particularly active against human mitochondrial ClpP (grey box). Fragment 14 represents the minimal structural element required for ClpP activation and deregulation towards unregulated proteolysis, although removing the macrocycle reduces potency greatly. Fragment 2a binds to a yet unknown binding site at mycobacterial ClpP and does not interfere with ClpX binding.

ADEP acts well against growing bacteria but does not require active growth to display its effects. Exceptional activity was reported against stationary *S. aureus* cells. While classical antibiotics lacked activity against a stationary *S. aureus* culture over a period of 5 days, an improved congener, ADEP4 (Figure 3), caused a 4 log_10_ reduction in viable cells on the second day, and pairing ADEP with classical antibiotics led to complete eradication (Conlon et al., 2013). In combination with linezolid, ADEP4 was effective against high-density stationary cultures of an extremely multi-drug resistant BORSA (borderline-oxacillin resistant *S. aureus*) strain and a VISA (vancomycin-intermediate *S. aureus*) strain. For both strains, the drug combination achieved 5–6 log_10_ reduction in colony-forming units (CFU) over 72 h (Mroue et al., 2019). Potent bactericidal activity was also achieved against stationary-phase vancomycin-resistant *E. faecalis* (VRE) by ADEP4 in combination with a variety of clinically approved antibiotic drugs, reducing CFUs by 5 log_10_ over 72 h (Brown Gandt, et al., 2018).

Anti-biofilm potential was also tested. Again, the activity of ADEP4 surpassed all classical antibiotics tested and, in combination with rifampicin, was able to eradicate a biofilm formed by an osteomyelitis-associated *S. aureus* strain to the limit of detection (Conlon et al., 2013). In another study with a mature MRSA biofilm, ADEP alone was able to eradicate all viable cells (Scheper et al., 2021). Against a mature enterococcal biofilm, ADEP4, in combination with partnering antibiotics, was superior to the standard combination therapies ampicillin-gentamycin and ampicillin-daptomycin (Brown Gandt et al., 2018). Furthermore, a biofilm produced by an *E. faecium* VRE strain isolated from an immunocompromised patient with enterococcal bacteremia, proved resistant to linezolid (50 μg/ml), daptomycin (50 μg/ml), and vancomycin (256 μg/ml), but was already susceptible against ADEP4 already at 0.2 µM (Honsa et al., 2017). Encouraging is the report that new analogs of the ureadepsipeptide series maintain the generally very good antibacterial potency of the compound class. UDEP16 (Figure 3) demonstrated the same potency in combination with rifampicin as ADEP4 and led to a 5 log_10_ reduction of viable cells in an *S. aureus* biofilm (Griffith et al., 2019). In the UDEP series, the α,β-unsaturated bond typical for potent ADEPs from previous series is replaced by urea, leading to increased metabolic stability (Griffith et al., 2019).

The activity of ADEP is also well-studied for ClpP from *M. tuberculosis*. Here, the ClpP tetradecamer is heteromeric and consists of one ClpP1 homo-heptamer and a distinct ClpP2 homo-heptamer, which stack back-to-back to form the functional proteolytic core (Akopian et al., 2012; Schmitz and Sauer 2014a). To assemble into a catalytically competent mixed tetradecamer *in vitro*, the purified proteins had to be exposed to certain N-terminally blocked dipeptides serving as agonists by binding to and aligning the active sites but resisting degradation (Akopian et al., 2012; Schmitz and Sauer, 2014a; Schmitz et al., 2014b). The requirement of these agonists is a characteristic feature of *M. tuberculosis* ClpP, and the molecular explanation is offered by a recent biochemical and biophysical study (Vahidi et al., 2020). Cryo-EM of *apo* and ADEP-bound ClpP1P2 from *M. tuberculosis* revealed the lack of a typical β-sheet within the handle region of ClpP (i.e., next to α5), making this region more flexible. The binding of the N-blocked dipeptide agonist increases the level of order and allows ClpP1P2 to shift from an inactive compact conformation to the extended state with a properly aligned catalytic triad (Vahidi et al., 2020). Within the cell, peptide products are assumed to serve this agonistic function, generated while a Clp-ATPase actively threads a protein into the proteolytic core (Schmitz et al., 2014b). ADEP alone could not shift *M. tuberculosis* ClpP to the catalytically competent state, but ADEP activated the peptide-agonist preconditioned complex further (Schmitz et al., 2014b; Famulla et al., 2016). In the presence of both types of activators, *M. tuberculosis* ClpP1P2 degraded 10-mer and 11-mer peptides, a branched peptide, and casein (Schmitz et al., 2014b; Famulla et al., 2016) but not the cell division protein FtsZ, neither *in vitro* nor in *M. tuberculosis* cells (Famulla et al., 2016). In an ADEP-ClpP1P2 co-crystal structure, ADEP occupied the H-pockets of ClpP2 exclusively (Schmitz et al., 2014b). ClpX and ClpC1 also address only ClpP2, and both Clp-ATPases were efficiently displaced by ADEP in competition experiments (Schmitz et al., 2014b; Famulla et al., 2016).

In contrast to most other bacterial species investigated to date, the Clp protease system of *M. tuberculosis* is essential for survival under all growth conditions, and this refers to the ClpP1 and ClpP2 paralogs as well as the cooperating Clp-ATPases ClpC1 and ClpX (Sassetti et al., 2003; Griffin et al., 2011; Ollinger et al., 2012; Raju et al., 2012; Roberts et al., 2013). This constellation forms the basis for the unusual killing mode of ADEP in this species. A conditional *Mycobacterium bovis* BCG mutant was constructed, a species in which *clpP1* and *clpP2* have 100% sequence identity to *M. tuberculosis.* Downregulation of the ClpP1P2 level in this strain enhanced the antibacterial activity of ADEP substantially. Increased antibiotic activity upon target downregulation is a clear indication of an *inhibitory* mechanism, demonstrating that ADEP does not kill *M. tuberculosis* by activating the ClpP1P2 core towards independent proteolysis of non-native substrates. In contrast in this species, where ClpP is essential, it is the interference with the natural functions of the Clp protease that causes cell death in the presence of ADEP (Figure 2). When ADEP binds to the H-pocket, the interaction of the Clp-ATPases with the ClpP core is blocked and it is presumed that this leads to the accumulation of toxic transcription factors (Raju et al., 2014; Famulla et al., 2016; Yamada and Dick, 2017). In a recent study, the activity of a range of synthetic ADEP fragments was explored against *M. tuberculosis* ClpP1P2. The authors hypothesized that the fragments might bind to the H-pocket and act similar to ADEP, thereby showing an inhibitory mechanism. While this was the case for some of the fragments (e.g., fragment 14; Figure 3), some other fragments demonstrated activating activities. For the latter group, this speaks against their binding to the H-pocket and implies that depending on the fragment structure more than one binding site at the ClpP barrel can be targeted (Schmitz et al., 2020). Fragment 2a (Figure 3) is an example of an activating fragment, that did not displace mycobacterial ClpX from ClpP but stimulated GFP-ssrA degradation by ClpXP. This finding suggests that fragment 2a does not compete with ClpX for the H-pocket but addresses a yet unknown binding site at ClpP (Schmitz et al., 2020). Exploring the chemical space of ADEP fragments is warranted as all full-size ADEP congeners tested so far showed only moderated MIC values against *M. tuberculosis* (Ollinger et al., 2012; Famulla et al., 2016; Schmitz et al., 2020).

With a molecular mass in the range of 700–900 g/mol and a hydrophobic nature, it is to be expected that ADEP congeners have difficulties in crossing the outer membrane of Gram-negative bacteria. Indeed, while isolated ClpP from *E. coli* was highly susceptible to ADEP and could be dysregulated in the same manner and with the same conformational characteristics as ClpP from *B. subtilis* or *S. aureus* (Kirstein et al., 2009a; Li et al., 2010), the MIC of ADEP against wildtype *E. coli* is high (>64 μg/ml). Using an *acrA* deletion mutant of *E. coli* and additionally, an outer membrane permeabilizing agent, an MIC of 3 μg/ml could be achieved (Brötz-Oesterhelt et al., 2005), indicating that the hurdle is not target- but uptake-related. *Neisseria* cells are more permeable than *E. coli* to a range of agents, and also the ADEP class demonstrated better activities against *Neisseria.* Promising activity was achieved with ADEP26 (Figure 3), designated ADEP-14 in a follow-up study from the same team (Goodreid et al., 2016; Binepal et al., 2020). The compound inhibited the growth of a diverse collection of clinical isolates (8 *N. meningitidis* strains, 14 *N. gonorrhoeae* strains) at 0.04 μg/ml (Binepal et al., 2020).

*Wolbachia* are also α-Proteobacteria, although with an atypical cell envelope. They are obligate endobacteria and reside as symbionts in the gut of parasitic filarial nematodes. Filarial infections can be treated with antibiotics because the worms depend on *Wolbachia* to survive. Several ADEP derivatives were evaluated for their anti-filarial activity (Schiefer et al., 2013). Among those, the natural product ADEP1 was the most effective congener in inhibiting *Wolbachia* residing in insect cells, and the compound demonstrated efficacy comparable to the gold standard doxycycline. When *Wolbachia* were targeted within filarial worms, ADEP2 surpassed ADEP1, probably due to its higher metabolic stability (Schiefer et al., 2013). *In vitro* casein degradation assays with purified recombinant *Wolbachia* ClpP confirmed activation by ADEP, and FtsZ degradation could be monitored by immunofluorescence microscopy of *Wolbachia* within insect cells (Schiefer et al., 2013).

In recent years, first *in vitro* studies on recombinant heteromeric ClpP1P2 complexes from additional pathogens have been performed, and the responsiveness of those new proteolytic cores to ADEP was characterized. *Chlamydia trachomatis* encodes two ClpP paralogs in two separate genetic loci, and the proteins emerge as heptamers after purification. Catalytic activity is only observed in the presence of a mixed ClpP1P2 hetero-tetradecamer, and the catalytic triads of both ClpP proteins jointly contribute to catalysis (Pan et al., 2019). As expected, ClpP1P2 alone can only degrade peptides, and in line with its established mechanism, ADEP allows chlamydial ClpP1P2 to degrade casein. ADEP and ClpX from chlamydia bind preferentially to the H-pocket of ClpP2 (Pan et al., 2019). *Clostridium difficile* also encodes two *clpP* genes in separate locations but in this case, hetero-tetradecamer formation did not occur *in vitro* (Lavey et al., 2019). Instead, ClpP1 formed a robust homomeric peptidase, which could be stimulated by ADEP and ClpX towards the degradation of a fluorogenic decapeptide and GFP-ssrA, respectively. ClpP2 did also act independently but was only very weakly active *in vitro*. Homomeric ClpP2 responded to ClpX stimulation but not significantly to ADEP (Lavey et al., 2019).

A ClpP1P2 complex was also described for Leptospira, the causative agent of an emerging zoonotic disease, and casein degradation was stimulated by ADEP (Dhara et al., 2021). In *Leptospira*, trigger factor (TF) is chromosomally colocalized with ClpP and ClpX, and the same applies to *E. coli* and many other organisms. This observation led two research teams to test a potential functional connection (Choudhury et al., 2021; Rizzolo et al., 2021). The addition of TF to *Leptospira* ClpXP stimulated casein degradation by the protease (Choudhury et al., 2021). In the *E. coli* study, proteolytic activation of ClpXP by Tig was demonstrated for a broader range of protein substrates *in vitro*, and from pulse-chase experiments in *E. coli* cells it was estimated that about 2% of newly synthesized proteins are degraded in a TF-dependent manner (Rizzolo et al., 2021). Peptide array mapping, mutagenesis and NMR analyses jointly support an interaction model which involves all three domains of TF and the AAA+ plus zinc-binding-domain of ClpX, establishing TF as a new adapter for ClpX (Rizzolo et al., 2021). However, TF also impacts the activity of the ClpP core when ClpX is absent (Choudhury et al., 2021). In *Leptospira*, TF stimulated the peptidase activity of ClpP1P2 and also casein degradation by the ADEP-activated *Leptospira* ClpP1P2 core (Choudhury et al., 2021). As demonstrated by these examples, ADEP is also increasingly used as a research tool to better characterize new ClpP homologs.

### Therapeutic Potential of the Acyldepsipeptide Class Against Bacterial Infections

Remarkable *in vivo* potency was reported for a range of ADEP derivatives in distinct bacterial infection models. In the treatment of an acute *E. faecalis* murine septicemia, a single dose of ADEP4 (0.5 mg/kg) or ADEP2 (1 mg/kg) was sufficient to ensure survival, and both ADEPs surpassed the efficacy of linezolid (Brötz-Oesterhelt et al., 2005). In another murine *E. faecalis* septicemia study, ADEP4 alone was as effective as ampicillin, the current clinical standard of care, and both antibiotics in combination were significantly more effective than either drug alone, with an additional 2 log_10_ reduction of the bacterial burden in the kidney compared to either monotherapy (Brown Gandt et al., 2018). An acute lethal *S. aureus* bacteremia was cured with 6 mg/kg ADEP4, while linezolid achieved only 60% survival at 12 mg/kg (Brötz-Oesterhelt et al., 2005). The ADEP derivative B315 (Figure 3) was more effective than vancomycin in reducing the bacterial load in the spleen and liver of mice acutely infected with *S. aureus* (Arvanitis et al., 2016). Also during the treatment of a lethal *S. pneumoniae* sepsis in rats, ADEP4 surpassed linezolid (Brötz-Oesterhelt et al., 2005).

Not only acute infections were successfully treated with ADEP. A rare and characteristic feature of ADEP is the potential to kill not only actively replicating bacteria but also persistent and dormant bacteria, biofilm-forming isolates, and bacterial cultures at high densities, conditions where standard antibiotics fail. The medical need for therapeutic options against infections by bacteria in such a resting physiological state is extremely high. There is no convincing treatment to date for e.g., endocarditis, osteomyelitis, and device-associated infections (Mroue et al., 2019). In a mouse model of a complicated thigh infection that emulates a deep-seated *S. aureus* infection in the immunocompromized patient, a combination of ADEP plus rifampicin led to sterilization of the infected tissue within 24 h (Conlon et al., 2013).

In a recent study aiming to evaluate the pharmacological potential of the ADEP class, ADEP4 was tested against an expanded panel of current multidrug-resistant staphylococci (methicillin- and vancomycin-resistant) and enterococci (VRE). Very promising MIC_50_ and MIC_90_ values (i.e., the lowest concentrations required to inhibit the growth of 50% and 90% of the strains from the panel, respectively) were obtained, and no preexisting cross-resistance among the bacterial population was detected (Mroue et al., 2019): MIC_50_/MIC_90_ (µg/ml) for *E. faecalis* (0.015/0.03), *E. faecium* (0.015/0.03), *S. aureus* (0.5/1). An *in vitro* pharmacokinetic and pharmacodynamic (PK/PD) study was also performed, using the dynamic hollow-fiber model. This is a technical system, where porous fibers are bundled within a cartridge, and bacteria occupy the space surrounding the fibers, being exposed to the fluid that leaks from them. By means of a set of hydrostatic pumps, nutrient broth of rising or declining antibiotic concentration is pumped through the fibers and equilibrates with the bacteria-containing compartment, simulating antibiotic concentrations in a time-controlled manner. Clinically achievable compound concentrations and their dynamic changes over time were simulated based on available pharmacokinetic data in human (for approved antibiotics) or animals (ADEP), and again, the potency of ADEP4 combinations was assessed against high-density (10^10^ Cfu/ml) cultures of *S. aureus* (MSSA and MRSA) and *E. faecalis* (VRE). Here, the combination of ADEP4 with bactericidal antibiotics proved highly effective, leading to an 8 to 9 log_10_ reduction in viable cells (Mroue et al., 2019). All available studies on the pharmacological evaluation of ADEP had a Gram-positive focus. Alternative antibiotic treatment options are also urgently needed against *Neisseria* due to the spread of high-level resistance among *N. gonorrhea* (Wi et al., 2017). As the MICs of certain ADEP derivatives against this species are low, further evaluation is also warranted against these Gram-negative bacteria.

For a toxicological assessment, few data are available so far. Histological analyses of kidney and liver sections from healthy mice treated with 50 mg/kg ADEP B315 did not indicate any significant tissue toxicity, in contrast to the kidney toxicity detected in mice treated with vancomycin (Arvanitis et al., 2016).

Regarding chemical and metabolic stability, substantial progress has been made within the ADEP class. Starting from the natural product ADEP1, which was susceptible to light and also metabolically unstable, thus lacking activity in infection models (Hinzen et al., 2006), the mono-unsaturated series around ADEP4 represented already a marked improvement, yielding a potent drug lead with excellent *in vivo* efficacy. Nonetheless, ADEP4 was still a high clearance drug (Brötz-Oesterhelt et al., 2005). With the discovery of the ureadepsipeptide series, metabolic stability has now substantially improved. During *in vitro* incubation with mouse liver microsomes, the compound half-life increased from 0.15 h for ADEP4 to 1.72 h for UDEP16, while retaining the same excellent target affinity, binding pose at ClpP and antibacterial activity (Griffith et al., 2019). This achievement is an important step towards a clinical candidate.

Another point to consider when discussing potential future ADEP therapy is the risk of emergence of ADEP-resistant mutants. In experiments to assess the degree of spontaneous resistance, a high mutation rate in the range of 10^–6^ was observed *in vitro* for several species of Firmicutes because *clpP* is not essential in these organisms under non-stressed growth conditions in the laboratory (Brötz-Oesterhelt et al., 2005; Conlon et al., 2013; Carney et al., 2014b; Brown Gandt et al., 2018; Malik et al., 2020). Mutants were also observed when high-density cultures were exposed to ADEP. In such experiments, an initial phase of strong bactericidal ADEP activity was followed by a phase of regrowth mediated by *clpP* mutants (Brown Gandt, et al., 2018; Mroue et al., 2019). However, ClpP is a stress protein with multiple functions in stress management in bacterial cells (Frees et al., 2014; Elsholz et al., 2017). The fact that *clpP* is strongly expressed when bacterial cells encounter protein stress implies that the unstressed conditions used commonly to determine resistance rates in the laboratory might not reflect the real situation that *clpP* mutants encounter in the host. Besides, the role of ClpP as a regulator essential for the expression of many virulence factors is established in a variety of bacterial species, and *clpP* deletion strains of many species were shown to be attenuated in the infection process (for reviews see Brötz-Oesterhelt and Sass, 2014; Frees at al., 2014; Culp and Wright, 2017; Bhandari et al., 2018; Moreno-Cinos et al., 2019b). In a recent study, the molecular defects of a collection of *clpP* mutants selected under ADEP pressure were investigated (Malik et al., 2020). In most, if not all mutants, the Clp protease system seemed out-of-function, as the mutations affected the catalytic function, oligomer dynamics or inter-subunit interactions. Even in cases where “only” the ADEP binding site (i.e., the H-pocket) was mutated, the interaction of the Clp-ATPases with the proteolytic ClpP core was impaired, also resulting in an out-of-function condition (Malik et al., 2020). Therefore, it is reasonable to assume that *clpP* mutations might occur with reduced frequency during ADEP therapy in the patient, as maintenance of functional ClpP is more important in the host. Nonetheless, any resistant mutant emerging under therapy is one too many. Therefore, it is highly recommended to apply ADEP only in combination with another antibiotic with proven efficacy against the target pathogen. In the preclinical investigations so far, various established antibiotic drugs showed potential as possible combination partners, offering some options (Conlon et al., 2013; Brown Gandt et al., 2018; Mroue et al., 2019). Care should be taken, however, with antibiotics, against which *clpP* mutations were reported to reduce susceptibility, among them glycopeptides, daptomycin, and β-lactam antibiotics, although effects might be species- or even strain-specific (Shoji et al., 2011; Song et al., 2013; Baek et al., 2014).

Drug resistance can also occur by efflux. For some species, it was described that ADEP congeners are subject to efflux. In *E. coli*, an *acrA* deletion mutant showed an increased sensitivity to ADEP1, indicating that the compound is a substrate for the RND (resistance-nodulation-cell division superfamily) pump AcrAB-TolC (Brötz-Oesterhelt et al., 2005). In *Streptomyces lividans*, a mutant overexpressing the ABC (ATP binding cassette)-transporter SclAB was more resistant than the corresponding wildtype against the A54556 natural product complex secreted into the agar by the producer strain (Gominet et al., 2011). In *Streptomyces coelicolor*, the MIC of the des-methyl-analog of ADEP4 rose by a factor of 2, when the ABC transporter SCO1719 was overexpressed (Compton et al., 2015) and *Mycobacterium tuberculosis* was twofold more susceptible to the des-methyl-analog of ADEP2, when the efflux pump inhibitors reserpine or verapamil were added (Ollinger et al., 2012). Notably, an ADEP fragment, i.e., *N*-heptenoyl-difluorophenylalanine (Figure 3) representing the linker plus side-chain of ADEP 4, was capable of inhibiting the efflux of ADEP in *S. coelicolor* and *Mycobacterium smegmatis*, probably by competing with the full-length ADEP for the binding sites at the pump(s) (Compton et al., 2015). To our best knowledge, there is no report on the efflux of ADEP in Firmicutes. For the cases of efflux mentioned above, a systematic assessment of the structural elements that make ADEP more or less sensitive to it (i.e., structure-activity-relationship) is lacking.

### Dysregulation of Mitochondrial ClpP by Acyldepsipeptide

Human mitochondrial ClpP is nucleus-encoded, translated in the cytoplasm, and imported into mitochondria via an N-terminal targeting sequence to be removed in this process (Corydon et al., 1998; Yu and Houry, 2007). The same applies to ClpX, which is the only known Clp-ATPase in mitochondria (Corydon et al., 2000; Nouri et al., 2020). There is no cytoplasmic version of either of them. Similar to its function in bacteria, mitochondrial ClpP, together with mitochondrial ClpX, is responsible for protein quality control and homeostasis. Mitochondrial ClpXP also regulates oxidative phosphorylation, mitochondrial ribosome biogenesis and contributes to various cellular stress response pathways and signaling cascades (Voos 2013; Valera-Alberni and Canto, 2018). Although ClpXP is present in almost all eukaryotic cells, its importance and expression levels vary among cell-types (Bross et al., 1995). In skeletal and heart muscle cells, ClpXP is strongly expressed, and ClpP-deficient muscle cells showed impaired proliferation, differentiation failure, and severely disturbed mitochondrial respiration (Bross et al., 1995; Deepa et al., 2016). In the lung, kidney, brain, and placenta, expression is significantly lower (Bross et al., 1995; Wong et al., 2018). Mutations in mitochondrial *clpP* are linked to type 3 Perrault syndrome, a rare human autosomal recessive condition, causing ovarian dysfunction in females and sensorineural hearing loss in both males and females (Gispert et al., 2013; Jenkinson et al., 2013; Brodie et al., 2018). In recent years, ClpP gained interest as an anticancer drug target, as it proved to be important for tumor proliferation in cancer types that have an increased dependence on mitochondrial function and thereby, high ClpP levels (Bhandari et al., 2018; Nouri et al., 2020). For instance, ClpP overexpression was noted in subgroups of patients with certain subtypes of acute myeloid leukemia, non-small cell lung cancer, sarcomas, as well as prostate, lung, liver, ovary, bladder, uterus, stomach, testis, and thyroid tumors (Cole et al., 2015; Nouri et al., 2020).

Due its potential to dysregulate bacterial ClpP, ADEP was also explored against human mitochondrial ClpP. As with the bacterial homologs, ADEP binds to the H-pockets of human mitochondrial ClpP, widens the entrance pores to the catalytic chamber, and activates the proteolytic core towards independent casein degradation (Lowth et al., 2012; Wong et al., 2018). Again, like with the bacterial counterparts, ADEP efficiently displaces human ClpX from ClpP, thereby inhibiting all the natural functions of the mitochondrial Clp protease (Wong et al., 2018). Among a series of synthetic ADEP derivatives tested, certain congeners with particularly good activation of mitochondrial ClpP, e.g., ADEP28 and ADEP-41 (Figure 3), were selected for mechanistic investigations on immortalized cell lines. The growth of several immortalized cell lines treated with those derivatives was inhibited with IC_50_ values of ∼0.5 µM (Wong et al., 2018). CLPP^−/−^ HEK293 cells were resistant to ADEP, and cells expressing more ClpP than the wildtype demonstrated increased sensitivity. From these data, it can be concluded that in HEK293 cells, the proliferation-inhibiting mechanism of ADEP is based on the activation and deregulation of the independent ClpP core and not on inhibition of the ClpX/ClpP interaction. Investigations on the phenotype of HEK293 cells treated with ADEP-41 indicated inhibition of oxidative phosphorylation, induction of mitochondrial fragmentation, fragmentation of chromosomal DNA, and activation of the intrinsic apoptotic pathway (Wong et al., 2018). In addition, ADEP1 was found to inhibit cell cycle progression in renal cancer cells (Xu et al., 2013), although high concentrations of the natural product had to be used (IC_50_ ∼50 µM). A molecular explanation was provided by the strong downregulation of cell cycle cyclin D1, a cyclin with multiple oncogenic functions that is commonly upregulated in cancers (Xu et al., 2013).

### Structure Activity Relationship of the Acyldepsipeptide Class

Several closely related natural product congeners were isolated from the culture broth of *Streptomyces hawaiiensis* NRRL 15010 (Michel and Kastner, 1985). “Factor A” (ADEP1, Figure 3) and “factor B” were the main components of the natural product complex and differ only in a single methyl group at the proline. The presence of the methyl moiety improves the antibacterial activity against *S. aureus* about 4-fold (Hinzen et al., 2006; Goodreid et al., 2014). MICs for ADEP1 were 4–6 μg/ml for *S. aureus*, 0.06–1.6 μg/ml for streptococci, and 0.4 for enterococci and set the benchmark for further improvement of the compound class (Brötz-Oesterhelt et al., 2005; Goodreid et al., 2014). Despite already promising MIC values, especially against streptococci and enterococci, ADEP1 lacked efficacy in infection models. Liabilities included moderate *S. aureus* activity, low chemical, and metabolic stability as well as insufficient solubility for parenteral application (Hinzen et al., 2006).

In an initial total synthesis campaign conducted at Bayer, several hundred derivatives were prepared and profiled (Brötz-Oesterhelt et al., 2005; Hinzen et al., 2006). Although molecular information on the target–compound interaction had not been available at that time, substantial improvement was achieved, as exemplified by ADEP2, ADEP4, and ADEP5 (Figure 3). Bis-fluorination of the phenyl ring in the linker strongly improved the antibacterial activity for staphylococci, streptococci, and enterococci, but only if 2 (no more, no less) fluorine atoms were introduced and if they were placed in positions 3 and 5 of the ring (Hinzen et al., 2006). Once the co-crystal structure became available, it became clear that this region reaches deeply into the H-pocket (Lee et al., 2010; Li et al., 2010), and 4-fluorination probably leads to a steric clash (Malik and Brötz-Oesterhelt, 2017). The Cα stereocenter at the Phe must be S-configured as R-configuration led to inactivity (compare ADEP3 in Brötz-Oesterhelt et al., 2005).

Rigidification of the macrolactone core enhanced activity further and was achieved by replacing the *N*-MeAla moiety with pipecolic acid. The crystal structure later showed that this region of the macrocycle is not submersed within the H-pocket but solvent-exposed (Lee et al., 2010; Li et al., 2010). Accordingly, the attachment of bulkier substituents was allowed at this position, for instance, to enhance solubility, as exemplified in ADEP5 (Brötz-Oesterhelt et al., 2005). A crosslinker could also be attached to assist mode of action studies (see ADEP6 in Brötz-Oesterhelt et al., 2005). Removal of the conjugated triene from the side-chain increased stability to temperature and light, and as long as the α,β-double bond was maintained and in *trans*-configuration, antibacterial activity remained very high. These modifications are exemplified by ADEP4, which became a drug lead with excellent *in vitro* and *in vivo* potency, as demonstrated by several studies described in this review. Reported MICs for ADEP4 were in the range of 0.05–0.2 μg/ml for *S. aureus*, 0.02 μg/ml for streptococci, and 0.008–0.1 μg/ml for enterococci (Brötz-Oesterhelt et al., 2005; Brown-Gandt et al., 2018; Griffith et al., 2019).

Further rigidification of the macrolactone core by the introduction of 4-methylpipecolate and *allo*-threonine were explored and led to ADEP1g (ADEP B315; ADEP-28) with even further improved antibacterial activity: MICs of 0.024 μg/ml were reported against *S. aureus* and ≤0.00002 μg/ml against *S. pneumoniae* and *E. faecalis* (Socha et al., 2010; Carney et al., 2014b; Arvanitis et al., 2016). Later, the dysregulating potential of the compound against human mitochondrial ClpP and its cytotoxic effects on eukaryotic cell lines were described, which occur at somewhat higher concentrations (IC_50_ ∼ 0.5 µM; Wong et al., 2018). Names in brackets indicate that the same compound was investigated in several studies and assigned different designations. Similar antibacterial and cytotoxic values were reported for ADEP1f (ADEP-41) with a MIC of 0.1 μg/ml against *S. aureus*, ≤0.00002 μg/ml against *S. pneumoniae* and *E. faecalis,* and an IC_50_ for eukaryotic cell lines at 0.5 µM (Carney et al., 2014a; Wong et al., 2018). In an attempt to increase stability, the ester linkage motif in the macrocycle was replaced by an amide or *N*-Me-amide but both modifications resulted in a strong decrease in activity (Li et al., 2017).

The side-chain offered some freedom for modification and was used as an important position for optimization. Replacing the α-carbon of the α,β-double bond by nitrogen yielded the saturated ureadepsipeptide series with improved metabolic stability and good potency. The MIC for UDEP16 against *S. aureus* was 0.1 μg/ml (Griffith et al., 2019). The acyl chain also tolerated further variations, but had to remain hydrophobic and within a certain length. In terms of activity, a linear heptenoyl side-chain was very good against staphylococci (ADEP4), but a branched chain was also allowed (Carney et al., 2015), and the introduction of a cyclohexane ring (ADEP2) and a *p*-methyl-phenyl moiety (UDEP16) were tolerated (Brötz-Oesterhelt et al., 2005; Hinzen et al., 2006; Griffith et al., 2019). Against Gram-negative bacteria and *Streptomyces* ssp., a lower degree of saturation seems beneficial. The triene natural product ADEP1 was more active than ADEP4 against *E. coli* and *Streptomyces*, although it has to be noted that the outer membrane of *E. coli* must be permeabilized to generate activity at all (Brötz-Oesterhelt et al., 2005; Thomy et al., 2019). A diene functionality is present in the natural product “factor D” and ADEP26, and both showed excellent activity against *Neisseria* (Goodreid et al., 2014; Goodreid et al., 2016; Binepal et al., 2020). The fact that both contain eight carbon atoms in contrast to the length of seven carbon atoms that was optimal for staphylococci suggests a somewhat longer binding pocket in Gram-negatives.

A fragment of ADEP4 consisting of linker and side-chain (*N*-heptenoyldifluorophenylalanine, fragment 14, Figure 3) represents the minimal requirement for obtaining antibacterial activity (MIC *B. subtilis* 8 μg/ml) and for triggering independent proteolysis of ClpP. Enzymatic assays suggested a similar binding mode as for full-length ADEP (Carney et al., 2014a). The peptidolactone macrocycle is inactive on its own but improves affinity and thereby potency by establishing additional contacts to the H-pocket.

## ClpP Activation and Dysregulation Beyond Acyldepsipeptide

**FIGURE 4:**
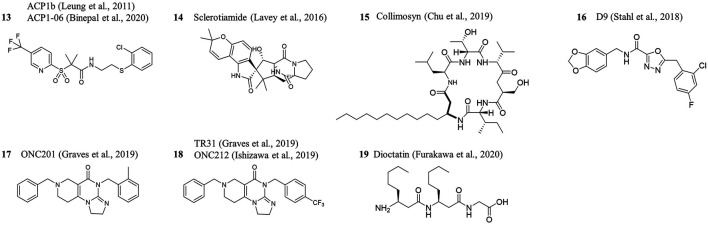
ClpP activators from other structural classes.

The observation that ClpP could serve as a druggable antibiotic target triggered interest in the search for structurally distinct activators of ClpP. In a high-throughput screening campaign, ∼65,000 compounds (synthetic chemicals, natural products, and marketed drugs) were tested for their potential to stimulate casein degradation by the *E. coli* ClpP core (Leung et al., 2011). A diverse set of non-ADEP compounds emerged, termed “activators of self-compartmentalizing proteases (ACP)”. ACP1b (Figure 4) was obtained by chemical modification of a screening hit and represents one of the best compounds from the initial study (Leung et al., 2011). Although certain ACPs showed, in principle, a similar activation mechanism as ADEP (i.e., stimulation of independent casein degradation by ClpP and stabilization of the tetradecamer), their dissociation constant K_D_ was substantially higher than for ADEP (Leung et al., 2011). In a follow-up study, a MIC value of 2–4 μg/ml against *N. meningitidis* was reported for the same compound, then termed ACP1-06, and an additional 100-fold improvement in MIC was achieved by another round of chemical optimization (Binepal et al., 2020). The compounds were active against multidrug-resistant *N. gonorrhoeae* and *N. meningitidis* isolates, showed no cross-resistance to established antibiotics, and killed *Neisseria* when residing inside eukaryotic cells (Binepal et al., 2020). Another screening approach, this time employing >20,000 bacterial and fungal extracts and ∼450 pure secondary metabolites, led to the indolinone natural product sclerotiamide (Figure 4) (Lavey et al., 2016). The compound activated *E. coli* ClpP to degrade casein and an undecapeptide but rather weakly compared to ADEP and less effectively than an ACP. Antibacterial activity against *E. coli* cells was not observed, and there was also no activation of *B. subtilis* ClpP (Lavey et al., 2016). Up to now, there is no information on the binding mode and molecular mechanism of sclerotiamide. In an alternative approach towards natural product-inspired bioactive agents, bioinformatic analyses were combined with chemical synthesis. From the genomic information of 96 non-ribosomal peptide synthetase gene clusters, structures of the putative natural products were deduced and 157 cyclic peptides were prepared by total synthesis (Chu et al., 2020). Nine of those showed antibacterial activity against diverse species and were characterized further. One peptide, inspired by a gene cluster of *Collimonas fungivorans* and thus termed collimosyn (Figure 4), inhibited the growth of *B. subtilis, S. aureus, and E. faecalis* (MIC of 4–8 μg/ml) and affected the proliferation of HELA cells (IC_50_ ∼8 μg/ml). To obtain first insight into the mode of action, resistant mutants were generated. Those contained out-of-function mutations in *clpP*, and a *S. aureus* Δ*clpP* strain was also insusceptible (MIC >128 μg/ml), suggesting that collimosyn kills *S. aureus* by ClpP activation and deregulation. *In vitro* experiments to confirm ClpP binding or activation have not been conducted so far (Chu et al., 2020).

For mitochondrial ClpP, further activators/deregulators were also described. Compound D9 (Figure 4) activated human mitochondrial ClpP to degrade casein and showed unusual selectivity by failing to induce proteolysis by the ClpP proteins of *E. coli*, *S. aureus*, and *Listeria monocytogenes* (Stahl et al., 2018). The interaction of human mitochondrial ClpX with ClpP was inhibited. The activation profile and potency of D9 were comparable to a compound resembling the ADEP4 linker and side chain. A crystal structure confirmed binding of D9 to the H-pocket, proved pore widening, and presented human mitochondrial ClpP in a compact conformation like it had been seen before with ADEP28 for this protein (Stark et al., 2018; Wong et al., 2018). Based on structure-activity analyses and modeling, it was proposed that the halogenated benzyl moiety of D9 and the bis-fluorinated phenylring of ADEP occupy the same position deep within the H-pocket.

Human mitochondrial ClpP was recently also identified as the molecular target of a new anticancer compound (Graves et al., 2019; Ishizawa et al., 2019; Wang and Dougan, 2019). ONC201(Figure 4) is the first-in-class member of the imipridone family of anticancer drugs and is currently being tested in clinical trials to treat diverse solid and hematologic tumors. Phase II clinical studies with positive outcomes were reported for refractory solid tumors (Stein et al., 2017) and glioblastoma, indicating that ONC201 can pass the blood-brain barrier (Arrillaga-Romany et al., 2017). ONC201 demonstrated substantial activity as a single agent and synergy in combination with other anticancer drugs (Prabhu et al., 2020). Preclinical investigations demonstrated enhanced activity of newer analogs from the same class (e.g., ONC212, alternative name TR31; see Figure 4) and suggest broad applicability to diverse types of cancer (Wagner et al., 2018; Aminzadeh-Gohari et al., 2020; Bonner et al., 2020; Jacques et al., 2020; Zhang et al., 2020). ONC206 has just entered Phase I clinical trials as a single agent for the treatment of central nervous system tumors (Bonner et al., 2020). Although other biological activities were also described for ONC201 and its congeners, e.g., dopamine D2 receptor antagonism or upregulation of the endogenous TNF-related apoptosis-inducing ligand (TRAIL) (Allen et al., 2015; Kline et al., 2018), a recent meta-analysis across 539 human cancer cell lines identified ClpP as the most significant biomarker for imipridone susceptibility of eukaryotic cells (Bonner et al., 2020). Two independent studies established ClpP as the primary target of the imipridones. In one of them, the authors started with the drug, immobilized an ONC201 derivative, and fished ClpP as the target (Graves et al., 2019). In the second study, the authors took the opposite approach. They screened a small library of drugs for the potential to stimulate casein degradation by human mitochondrial ClpP and found ONC201 as a screening hit (Ishizawa et al., 2019). Collectively the two studies established the following line of evidence. Only CLPP^+/+^ and not CLPP^−/−^ cells were sensitive to the imipridones, and ClpP expression levels in cancer cells directly correlated with their sensitivity to the drugs. A single amino acid substitution in ClpP led to ONC201 resistance and expression of wildtype ClpP in resistant tumor cells restored sensitivity (Graves et al., 2019; Ishizawa et al., 2019). On the molecular level, the mechanism of ONC201 corresponds to that of ADEP. A co-crystal structure of ONC201 and human mitochondrial ClpP demonstrated binding to the hydrophobic pocket (Ishizawa et al., 2019). As a result, a stable active tetradecamer was formed with widened entry pores capable of degrading larger peptides and casein. In cancer cells, ClpP activation by ONC201 led to the degradation of respiratory chain subunits, impaired oxidative phosphorylation, and apoptosis. Despite the same principle mechanism, the imipridones were much more potent than ADEP1 in activating human mitochondrial ClpP and in inhibiting the proliferation of cancer cells (Ishizawa et al., 2019).

The mitochondrial Clp protease of the fungus *Aspergillus flavus* was recently identified as the target of the bacterial natural product dioctatin A (Furukawa et al., 2020). The compound was identified in a search for inhibitors of aflatoxin production in the fungus, but the mechanism was elusive. Also here, affinity chromatography was conducted, using immobilized dioctatin, a simplified analog of the natural product amenable to total synthesis. ClpP bound selectively to the compound. Dioctatin stimulated *A. flavus* ClpP *in vitro* for independent casein degradation, although 20 µM dioctatin had to be applied to demonstrate an appreciable proteolytic effect, compared to 1 µM ADEP1. Proteomics analysis of a mitochondrial extract digested by dioctatin-activated mitochondrial ClpP *in vitro* demonstrated truncated fragments of several energy-related mitochondrial proteins. Consistently, a variety of changes in energy metabolism were noted as well as reduced histone acetylation, the latter causing reduced expression of the aflatoxin biosynthesis genes (Furukawa et al., 2020).

In a recent publication the term “paracatalytic inducers” was coined for agents that accelerate an enzyme reaction that is not physiological (Callahan et al., 2020). The ClpP activators discussed in this review promote paracatalysis of the “substrate ambiguity” subtype, which refers to agents that enable the transformation of non-native substrates. It seems that more and more paracatalytic inducers of ClpP emerge, now that people have started looking for them.

## Conclusion

Since the discovery of ClpP as the target of ADEP about 15 years ago, our knowledge base has substantially expanded. On the one hand, on the compound class of ADEP itself, concerning the pharmacophore and structure-activity-relationship responsible for the biological activities, as well as on the multifaceted mechanism of action. ADEP exerts elaborate conformational control over the entire ClpP tetradecamer, leading to allosteric activation and pore opening to allow unchecked degradation of non-native substrates. A wealth of information also accumulated on ClpP itself, including its role as a major stress protein, virulence factor, and global regulator.

ClpP emerged as a prime antibacterial, anticancer, antiplasmodial, and antifungal target, ubiquitous across organisms and druggable by diverse structural classes. Here, ADEP often served as a forerunner in target validation studies. Nowadays, in mode of action discovery studies for new agents (such as collimosyn or ONC201), ClpP activation is already regularly taken into account and tested as a potential growth inhibitory mechanism. Interesting about ClpP, and also unusual, is that both ClpP activation and inhibition have therapeutic potential. ClpP activation is appealing as active growth of cells is not required, which holds promise for the treatment of dormant cells and persistent infections. ClpP inhibition is also promising; however, this is a broad topic and was therefore omitted from this review. Briefly, in bacteria, ClpP inhibition represents a broad-spectrum anti-virulence approach. In eukaryotic cells, it is encouraging that ClpP inhibition causes only a mild phenotype in most cell types while certain cancer subtypes with enhanced dependence on ClpP are highly sensitive.

The ADEP compound class is close to generating a clinical candidate, and from the imipridone class, two candidates are already being tested in clinical trials. Although both compound classes act by the same mechanism and target the same binding site at ClpP, ADEP is particularly potent in bacteria, whereas the imipridones, from all of what is published to date, clearly surpass ADEP in cancer cells. Compound D9 was even selective for human mitochondrial ClpP. It will be interesting to understand the underlying molecular interactions better and exploit them for the development of even more selective agents.

ADEP also proved to be a handy tool. Before Clp-ATPase/ClpP-Clp structures were accessible by cryo-EM, the ADEP-ClpP co-crystal structures of various organisms allowed detailed insight into the operation mode of ClpP and the functionality of the H-pocket as a master-regulator for controlling the conformation of ClpP along the vertical and horizontal axis. More and more research groups interested in new ClpP proteins with low activity after purification employ ADEP for stabilizing ClpP in an active conformation during their *in vitro* experiments. In some bacteria and when applied in concentrations close to the MIC, ADEP is also instrumental for studying cell division processes, capitalizing from the fact that FtsZ is a preferred target of ADEP-activated ClpP under these conditions.

The ADEP class originates from a natural product complex. Nature has employed ClpP as a target long before we discovered it, highlighting the value of mode of action studies with natural products.
